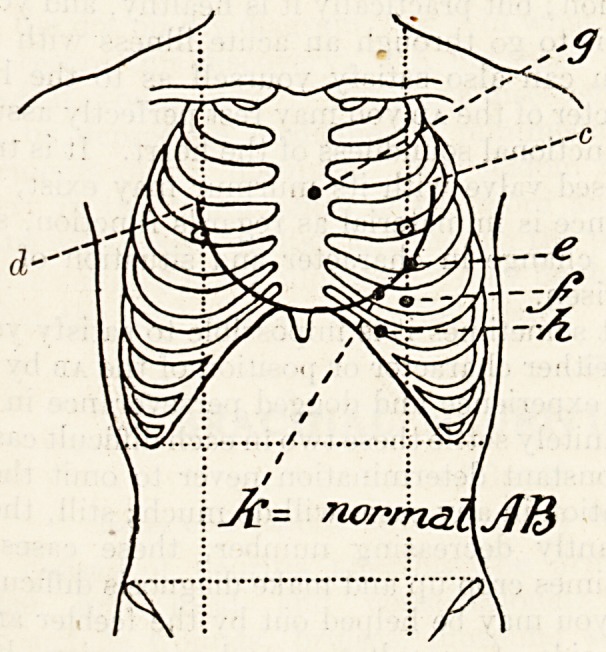# The Importance of Localising the Heart's Apex Beat

**Published:** 1907-11-23

**Authors:** Arthur Foxwell

**Affiliations:** Senior Physician to the Queen's Hospital, Birmingham.


### HOSPITAL CLINICS.

### THE IMPORTANCE OF LOCALISING THE HEART'S APEX BEAT.

By ARTHUR FOXWELL, M.A., M.D.Cantab, F.R.C.P., Senior Physician to the Queen's Hospital, Birmingham.

I OWE much to the teaching of Sir Douglas Powell, perhaps most to his splendid example of careful accuracy of diagnosis; but what stands out with greatest distinctness in my recollection is his insistence that always the first thing to do in note-taking is to fix the apex beat with painstaking exactitude. I well remember when acting as his house physician at Brompton, after reading a long and, as I thought, excellent " present state " to him, he took the paper from me and quietly drew his pen through the whole of it, saying gently, " I told you to always start off with the sentence The heart's apex beats in such and such a place." I was wroth at the time, but he said: "You will thank me for it some day," and I believe there are few days of active clinical work when I have not felt grateful for his emphasis of this point.

Not only in chest cases, but in every case that comes to me as a general physician, do I adhere rigidly to his rule. Doing so has saved me much time and many mistakes. It has perhaps stood me in even better stead in cases of abdominal than in those of chest disease, and it has often helped me to diagnose between the results of intracranial disease due to vascular degeneration and those due to other causes.

In this paper I wish to indicate a few of the more evident things to be learnt from it. The most fixed portion of the heart is that which surrounds the aortic valves: it is very seldom indeed that any notable change of position is observed in this. On the surface of the chest the centre of this fixed area corresponds to the middle of the junction of the third left cartilage with the sternum (G). So long as the apex beat (AB) is formed by the left ventricle a line from G to the AB represents the extreme length of this ventricle, and any lengthening of this line indicates its dilatation, for an increase due to pure hypertrophy is so small as to be barely noticeable.

Hence, as I have shown elsewhere,[1] so long as the left ventricle remains normal dilatation of the right ventricle must push the AB up and out along the arc of a circle (DC), whose centre is G, and diameter the length of the normal left ventricle (GK); or, conversely, if the AB be thus altered in position it shows dilatation of the right ventricle only. For instance, if the AB move from its normal position [2] to the middle of the fourth space in the nipple line (E), then there is enlargement of the right ventricle only; but if it move out to the nipple line, still remaining in the fifth space (F), then both ventricles are about equally enlarged; and, thirdly, if, without travelling out, it descend to the sixth space (H), then the enlargement is almost or altogether that of the left ventricle.

As to its character: if it be like the blunt thrusting forwards of a small hemisphere and of long duration, there is hypertrophy. If it be a sharp slap or knock there is weakness, or dilatation (or both), and the ventricle is not emptying itself. If the impulse be not localised, but widely distributed over all the præcordia to the left of the sternum below the third cartilage, and a strong epigastric impulse be also felt, then the AB in all probability is the AB of the right ventricle, which is enlarged and hypertrophied, whilst the condition of the left ventricle is masked.

Suppose the AB be diffuse, feeble, and yet reaching farther to the left than the normal, as well as difficult to perceive, there is probably pericardial effusion, and the AB felt is merely the impulse given to the effusion by the true AB.

A similar AB may be experienced in a left-sided pleural effusion where the heart is fixed by adhesion and the pleural fluid comes between it and the chest wall. But usually in pleural effusion the heart is moved bodily towards the other side, and the AB points more or less directly downwards, occupying the sixth space close to the left edge of the sternum; or it may even occupy the fifth right space, but in this extreme case it is farther to the right than the cardiac base, the long axis of the ventricle pointing to the right instead of to the left.

In phthisis adhesion of the pericardium to the pleura over a cavity often causes remarkable movement of the AB, and the consequent dislocation of the heart is no inconsiderable factor in bringing on a fatal issue. In such cases of tuberculous cavitation one often learns more concerning the progress of the disease from carefully watching this movement of the AB than from any other single sign or symptom. I have seen as great cardiac translation from the gradual contraction of a large cavity in the left lung as from

[1] Bradshaw Lecture on Functional Heart Murmurs, 189[illegible].

[2] In the middle of the fifth costal interspace at the junction of the middle and outer thirds of a horizontal line drawn from the left edge of the sternum to meet a perpendicular drawn through the normally situated left nipple (or dropped from the junction of the middle and outer thirds of the left clavicle).

any effusion, however extensive. It is not even necessary for the pericardium to be adherent to the pleura, as atmospheric pressure obliges the heart to follow the movements of pulmonary contraction to a large extent.

The gradual masking of the AB in a doubtful case of thoracic disease is a sign of very sinister significance, pointing, as it does, to involvement of heart or pericardium in new growth.

I believe the AB is of great help in the diagnosis of "fatty degeneration" of the heart: the amount of force required to contract a cavity against a given pressure increases immensely with the size of the cavity. I cannot understand how heart muscle with any serious amount of fatty degeneration can exert force sufficient to contract such a cavity, and therefore put "fatty degeneration" on one side when dealing with a heart whose AB is much displaced outwards or downwards. On the other hand, a "fatty" heart has its AB nearly always displaced some half-inch to the left, in consequence of the heart taking on the globular form of least resistance (tonelessness).

A uniform enlargement of the liver does not displace the AB, so long as the liver is free to move downwards; if, then, in connection with hepatic trouble such displacement occur it is due to large local bulgings of the left lobe, and suggests new growth or hydatid. The same holds good with the spleen, except in those extreme cases where it presses against the pelvic rim. But if with either big spleen or liver there be localised peritonitis, or if there be subphrenic abscess, then the resulting adhesions may displace the AB.

Very great displacement may be due to tympanites, whether of stomach or bowel. I have noted the apparent AB in such cases to be in the third space, or even behind the third rib, and out beyond the nipple line: I say apparent because the true apex is probably behind the distended upper portion of the peritoneal cavity. Here, again, the position of the AB is a very good criterion of the amount of abdominal distension and of the urgency of any treatment required for its relief.

An acute and considerable distension of the stomach alone may so raise the left diaphragm that the heart is pushed to the right, and the AB becomes epigastric. This may be misleading, suggesting a dilated right ventricle and combating the idea of subdiaphragmatic distension, a suggestion usually easily dispelled by examination of the left lateral base of the thorax, though the condition found here may occasionally be confused with left basic pneumothorax.

In nervous debility and chronic renal disease the position of the AB may be the same, namely, in the fifth space and nipple line; but its character at once distinguishes the two conditions: in renal disease the impulse is one of hypertrophy; in debility it has the slapping feeble character of pure dilatation. Indeed, in woman this position and character of the AB is almost pathognomonic of renal disease: in man it is not so helpful, as we get the same AB in cases of degenerated arteries without renal disease; but in woman degenerated arteries are rare, and chronic high tension, with its resulting action on the AB, is nearly always due to renal degeneration.

The position of the AB is a great help in distinguishing between aortic valvular disease and aortic aneurysm. In aneurysm of the ascending arch the heart is pushed bodily out and down along the left anterior slope of the diaphragm. The AB thus takes up a position considerably to the left of its normal situation, and is only moderately depressed—*e.g.* it may be felt half an inch outside the nipple line beneath the sixth rib. Such a position is almost impossible if the case be one of aortic regurgitation only, the sixth space inside the nipple line being the usual situation. Even when aortic regurgitation is accompanied by considerable degeneration of the aorta with the accompanying widening and elongation of the vessel, still the AB never takes up the position it does in dislocation of a normal heart: the enlargement of the left ventricle always thrusts it too far down and too little out. Again, aneurysm by itself does not enlarge the heart; hence if with severe anginoid pains in the left chest and arm suggestive of an obscure aneurysm of the transverse or descending arch, you get an AB outside the nipple line in the fifth space accompanied or not by functional mitral regurgitation, then the probability is that the pain is due to the overtaxing of feeble muscle and not to aneurysm.

An AB quite normal in situation almost assures you that the heart is functionally healthy; there may be a sclerosed valve, some hypertrophy, or even slight fatty change, or partial pericardial adhesion; but practically it is healthy, and you may trust it to go through an acute illness with safety. If you can also satisfy yourself as to the healthy character of the AB you may rest perfectly assured of the functional soundness of the heart. It is true the sclerosed valve with its murmur may exist, but its existence is immaterial as regards function, so long as no change in character and situation of the AB has arisen.

But sometimes it is impossible to satisfy yourself as to either character or position of the AB by palpation: experience and dogged perseverance in trying to definitely settle these two in each difficult case, and the constant determination never to omit their examination in any case, will do much; still, though a constantly decreasing number, these cases must sometimes crop up and make diagnosis difficult. In such you may be helped out by the feebler and less exact aids of auscultation and percussion, but the knowledge they give is always lacking in the precision afforded by the more elementary sense.

This reminds me of another great advantage gained by fixing the AB: not only does it tell you so much concerning your patient, but it does this by a sense that never fails: sight and hearing may grow dull and lack discrimination, but the sense of touch can rarely go from both hands; if it ever should, you may be sure it is high time for you to give up the practice of clinical medicine, however keen your eyes and ears may be.

We must never rest content with merely "spotting" the AB—just putting one's finger on it and guessing its anatomical position from so doing; we may happen to be right: I have often proved myself to be wrong. Such a procedure is ludicrous from a scientific point of view; from that of our patient it is heinous. Let us always carefully examine for the true nipple line and the true left edge of the sternum, not always easy things to find; also and much more importantly must we always count the ribs to make sure that our "shot" of fourth, fifth, or sixth space or rib is a correct one. Unfortunately this counting is no easy matter, and unless we master it when young it will prove irksome; but it is absolutely necessary; even after all these years my "shot" is not always right, and I very rarely omit the counting; in fact, it is one of those acquired habits which are almost instinctive. I admit that here again it is sometimes impossible to be sure; even so simple a feat as counting five ribs correctly cannot always be done in clinical medicine. It is a good instance of how the complexity of the human body intensifies the difficulty of examination.

Finally, if I may venture to do so, I would advise every practitioner to take a little notebook with him, a diagram of the trunk on one side of each leaf, and on the other a few spaced headings for notes. I show you a sample of one such leaf. The diagram is the important part; if you fill in this it is wonderful how much more accurate it makes you: often after an examination, when I go to my diagram to fill in the results I find I have not examined with accuracy sufficient to delineate these, and have to re-investigate some point or other. Do not, when you try to fix the AB, rest content with saying to yourself: "Oh, here it is" (putting your finger on the spot): do this by all means, but mentally add, "where shall I place the dot on my diagram" or "in what terms shall I record this discovery." A big word for so small a feat, you may say. Yet it is a discovery: do not let us depreciate medicine by minimising the achievements of her disciples. Not seldom it is a discovery in the truest sense of the word and one pregnant with large results; for, though the patient may have suffered many illnesses, it may be the first time that the AB has been truly delineated. Your accurate investigation may place an entirely fresh complexion on the patient's condition: this true diagnosis of one particular point may alter the general diagnosis altogether—*e.g.* a case sent to me as one of aortic degeneration was shown to be acute endocarditis of the aortic valve from the position of AB.

Do not be content with placing the AB beneath a rib or in a space, but let it be middle, upper, or lower border of one of these. It will make all the difference to you in following out a case of, say, typhoid or phthisis. An AB may remain in the fifth space, and yet its shifting out and down to the sixth rib may indicate serious cardiac failure; not only an increasing muscle weakness, but a dilatation which greatly increases the difficulty of emptying the ventricles, and almost certainly indicates that this emptying is very imperfectly performed.

This is not the time to point out what change in the shape of the heart accompanies this change of AB; yet we know it takes place in all cases where the heart fails from pure muscle feebleness. The change is from a strenuous cone to a flaccid spheroid, and therefore its cavities are much larger than the mere increase in length of diameter would suggest. This we learn directly from percussion; but, as accumulated experience has shown that it is an invariable concomitant of the change of AB, we may justly infer it from an accurate estimation of this latter change alone. I say accurate estimation advisedly to remind you that as a matter of practice it is always necessary to use both forms of investigation so that one may correct or corroborate the other; for, alas! the best of us is a very imperfect examiner. Not, let me hasten to add, because our intellects are below the human average, but because the great mistress whom we have chosen to serve is so exceeding great and wonderful. "Age cannot wither nor custom stale her infinite variety."

## Figures and Tables

**Figure f1:**